# Hantavirus in Northern Short-tailed Shrew, United States

**DOI:** 10.3201/eid1309.070484

**Published:** 2007-09

**Authors:** Satoru Arai, Jin-Won Song, Laarni Sumibcay, Shannon N. Bennett, Vivek R. Nerurkar, Cheryl Parmenter, Joseph A. Cook, Terry L. Yates, Richard Yanagihara

**Affiliations:** * University of Hawaii at Manoa, Honolulu, Hawaii, USA; † Korea University, Seoul, Republic of Korea; ‡ University of New Mexico, Albuquerque, New Mexico, USA

**Keywords:** Hantavirus, shrews, Blarina brevicauda, phylogeny, dispatch

## Abstract

Phylogenetic analyses, based on partial medium- and large-segment sequences, support an ancient evolutionary origin of a genetically distinct hantavirus detected by reverse transcription–PCR in tissues of northern short-tailed shrews (*Blarina brevicauda*) captured in Minnesota in August 1998. To our knowledge, this is the first evidence of hantaviruses harbored by shrews in the Americas.

Rodents and their ectoparasites serve as reservoirs and vectors of myriad viruses and other pathogenic microbes. In contrast, the role of insectivores (or soricomorphs) in the transmission and ecology of zoonoses is largely unknown. Because some soricomorphs share habitats with rodents, shrews might also be involved in the maintenance of the enzootic cycle and contribute to the evolutionary history and genetic diversity of hantaviruses.

Hantavirus antigens have been detected in the Eurasian common shrew (*Sorex araneus*), alpine shrew (*Sorex alpinus*), Eurasian water shrew (*Neomys fodiens*), and common mole (*Talpa europea*) in Russia and the former Yugoslavia (*1*–*3*). More than 20 years ago, when Prospect Hill virus was discovered in meadow voles (*Microtus pennsylvanicus*) captured in Frederick, Maryland, USA, serologic evidence suggestive of hantavirus infection was found in the northern short-tailed shrew (*Blarina brevicauda*) (*4*). However, virus isolation attempts were unsuccessful, and molecular tools such as PCR were unavailable. Empowered by robust gene-amplification techniques and the complete genome of Thottapalayam virus (TPMV) isolated from the Asian house shrew (*Suncus murinus*) (*5*,*6*), we have identified a genetically distinct hantavirus in the northern short-tailed shrew.

## The Study

After obtaining approval from the University of Hawaii Institutional Animal Care and Use Committee, we retrieved lung and liver tissues of 30 northern short-tailed shrews captured from several regions within the United States during 1994–1999 (Table 1) from deep-freeze storage at the University of New Mexico Museum of Southwestern Biology. Total RNA was extracted from shrew tissues by using the PureLink Micro-to-Midi Total RNA Purification Kit (Invitrogen, San Diego, CA, USA). cDNA was then prepared by using the SuperScript III First-Strand Synthesis System (Invitrogen) for reverse transcription–PCR (RT-PCR) with oligonucleotide primers designed from TPMV and other hantaviruses: medium (M) (outer: 5′-GGACCAGGTGCADCTTGTGAAGC-3′, 5′-GAACCCCADGCCCCNTCYAT-3′; inner: 5′-TAAVTTCAMCAACATGTCT-3′, 5′-CATGAYATCTCCAGGGTCHCC-3′) and large (L) (outer: 5′-CAGTCWACARTTGGTGCAAGTGG-3′, 5′-TCCATKATWGACATBGMRCCA-3′; inner: 5′-YTMATGTATGTTAGTGCAGATGC-3′, 5′-GRTTAAACATACTCTTCCACATCTC-3′). For confirmation, RNA extraction and RT-PCR were performed independently in a laboratory in which hantaviruses had never been handled. Amplicons were sequenced directly by using an ABI Prism 377XL Genetic Analyzer (Applied Biosystems, Foster City, CA, USA).

**Table 1:** Reverse transcription–PCR detection of hantavirus sequences in tissues of *Blarina brevicauda*, United States

State	County	Trapping date	No. tested	No. positive
Indiana	Porter	Jul 1994	2	0
	Westchester	Jul 1994	1	0
Iowa	Allamakee	Aug 1994	5	0
Maryland	Charles	Sep 1997	3	0
Michigan	Benzie	Jul 1994	1	0
	Crawford	Jul 1999	2	0
Minnesota	Morrison	Aug 1998	12	3
Ohio	Summit	Jul 1994	2	0
Virginia	Appomatox	Jul 1994	1	0
	Page	Mar 1995	1	0

Of the 30 northern short-tailed shrews tested, hantavirus M-segment sequences were amplified from lung tissues of 3 of 12 animals captured in Camp Ripley (46.185°N, 94.4337°W), a 53,000-acre, state-owned military and civilian training center near Little Falls, in Morrison County, Minnesota, USA, in August 1998 (Table 1). Pairwise alignment and comparison of the 1,390-nt region (463 aa) spanning the Gn and Gc glycoprotein–encoding M segment indicated differences of 33.6%–41.9% and 32.7%–47.4% at the nucleotide and amino acid levels, respectively, from representative hantaviruses harbored by *Murinae*, *Arvicolinae*, *Neotominae*, and *Sigmodontinae* rodents (Table 2). No insertions or deletions were found in the regions sequenced compared with sequences of other hantaviruses. The new hantavirus, designated Camp Ripley virus (RPLV), showed sequence similarity of 98.1%–98.5% among the 3 strains.

**Table 2:** Nucleotide and amino acid sequence similarities of partial medium (M) and large (L) segments of Camp Ripley virus and other hantaviruses*

		M Segment			L Segment	
Virus	Strain	1,390 nt	436 aa		490 nt	163 aa
Hantaan	76–118	65.6	66.9		71.2	79.3
Soochong	SC-1	65.2	64.4		73.2	77.3
Dobrava	AP99	66.4	67.3		72.5	79.0
Seoul	HR80–39	65.8	67.1		71.3	77.7
Sangassou	SA14	62.6	58.7		69.3	78.8
Puumala	Sotkamo	60.1	55.0		71.6	72.3
Tula	M5302v	61.9	54.6		67.2	69.3
Prospect Hill	PH-1	58.1	52.6		NA	NA
Sin Nombre	NMH10	60.3	57.0		65.9	65.5
Andes	Chile 9717869	60.2	56.3		70.2	69.2
El Moro Canyon	RM97	59.8	56.5		NA	NA
Tanganya	Tan826	NA	NA		72.3	75.5

*Values are percentages. NA, not available.

Analysis of a 490-nt (163-aa) region of the L genomic segment amplified from 2 of the 3 shrews indicated that RPLV differed from rodentborne hantaviruses by 26.8%–34.1% at the nucleotide level and 20.7%–34.5% at the amino acid level (Table 2). RPLV differed from Tanganya virus (TGNV), a hantavirus detected recently in the Therese shrew (*Crocidura theresae*) in Guinea (*7*), by 27.3%–28.8% and 24.0%–25.0%, respectively. The higher degree of sequence similarity in the L segment between RPLV and other hantaviruses probably signifies the limits of functional preservation of the RNA-dependent RNA polymerase.

Repeated and exhaustive phylogenetic analyses based on nucleotide and deduced amino acid sequences of the M and L genomic segments generated by the maximum-likelihood method indicated that RPLV was distinct from rodentborne hantaviruses (with high bootstrap support based on 100 maximum likelihood replicates) (Figure). Similar topologies were consistently derived by using various algorithms and different taxa (including La Crosse virus) and combinations of taxa, which suggested an ancient evolutionary origin. However, definitive conclusions about the molecular phylogeny of RPLV and its relationship to TGNV and other soricidborne hantaviruses must await complete-genome sequence analyses.

**Figure:**
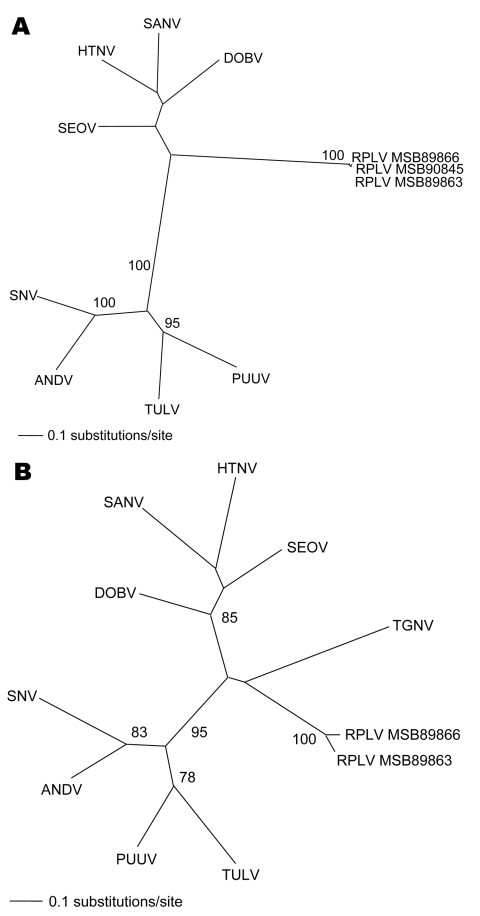
Phylogenetic trees generated by maximum likelihood method and generalized time reversible + I + G model of evolution as estimated from data on alignment of the partial (A) 1,390-nt medium (M)– and (B) 490-nt large (L)–genomic segments of Camp Ripley virus (RPLV). Phylogenetic positions of RPLV strains MSB89863, MSB89866, and MSB90845 are shown in relationship to representative Murinae rodentborne hantaviruses, including Hantaan virus (HTNV 76–118, NC_005219, NC_005222), Sangassou virus (SANV SA14, DQ268651, DQ268652), Dobrava virus (DOBV AP99, NC_005234, NC_005235), and Seoul virus (SEOV 80 39, NC_005237, NC_005238); Arvicolinae rodentborne hantaviruses, including Tula virus (TULV M5302v, NC_005228, NC_005226) and Puumala virus (PUUV Sotkamo, NC_005223, NC_005225); and Neotominae and Sigmodontinae rodentborne hantaviruses, including Andes virus (ANDV Chile 9717869, NC_003467, NC_003468) and Sin Nombre virus (SNV NMH10, NC_005215, NC_005217). Tanganya virus (TGNV Tan826, EF050454) from the Therese shrew (*Crocidura theresae*) is also shown. Host identification of *Blarina brevicauda* was confirmed by morphologic assessment of voucher specimens and by mitochondrial DNA sequences (data not shown). The numbers at each node are bootstrap support values (expressed as the percentage of replicates in which the node was recovered), as determined for 100 maximum likelihood iterations under the same model of evolution by PAUP version 4.0 (http://paup.csit.fsu.edu). The scale bar indicates 0.1 nt substitutions per site. GenBank accession nos. for RPLV: M (EF540774, EF540775, EF540773) and L (EF540771, EF540772).

## Conclusions

As we had previously encountered in sequencing the entire genome of TPMV (J.-W. Song, R. Yanagihara, unpub. data), the divergent genome of RPLV presented challenges in designing suitable primers for RT-PCR. We were also constrained by the limited availability of tissues from the 3 infected shrews and the need to retain small portions of tissues for future virus isolation attempts. Consequently, we have been hitherto unable to obtain the full-length sequence of RPLV.

The northern short-tailed shrew (family *Soricidae*, subfamily *Soricinae*), 1 of 2 poisonous mammals in North America (*8*), inhabits forests and grasslands within the central and eastern half of the United States, extending north to Canada, west to Montana, and south to Tennessee and Georgia. Cytochrome *b* mitochondrial DNA and 16S rRNA sequence analyses support a monophyletic origin for the genus *Blarina*, with phylogeographic structuring of northern short-tailed shrews into well-defined groups to the east and west of the Mississippi River basin (*9*). Current studies will examine whether RPLV is harbored by the eastern haplogroup of northern short-tailed shrews and by the southern short-tailed shrew (*Blarina carolinensis*), a closely related species, which inhabits the southeastern United States, extending as far north as southern Illinois and south-central Virginia and as far south as central Florida.

Given the sympatric and synchronistic coexistence of northern short-tailed shrews with *Neotominae* and *Arvicolinae* rodents (such as *Peromyscus leucopus* and *Microtus pennsylvanicus*) and their ferocious territorial behavior, hantavirus spillover may be possible. Viruses closely related antigenically to Hantaan virus have been isolated from the Asian house shrew (*Suncus murinus*), greater white-toothed shrew (*Crocidura russula*), and Chinese mole shrew (*Anourosorex squamipes*) (*10*–*12*), which suggests that shrews are capable of serving as incidental hosts for hantaviruses typically harbored by rodents.

Shrews that harbor genetically distinct hantaviruses pose a compelling conceptual framework that challenges the long-accepted dogma that rodents are the sole reservoirs of hantaviruses. Viewed within the context of the recent detection of TGNV in the Therese shrew in Guinea, the identification of RPLV in the northern short-tailed shrew in the United States indicates that renewed efforts, facilitated by the rapidly expanding sequence database of shrew-borne hantaviruses, will lead to the discovery of additional hantaviruses in soricids throughout Eurasia, Africa, and the Americas. Our preliminary studies indicate 3 other novel soricid-borne hantaviruses in the Republic of Korea, Vietnam, and Switzerland. To establish if >1 of these newly identified hantaviruses is pathogenic for humans will require development of robust serologic assays (*13*) and application of other sensitive technologies, such as microarray analysis (*14*,*15*), for rapid detection of shrewborne hantavirus RNA in human tissues and bodily fluids.
